# Enhancement of Anti-Tumoral Properties of Paclitaxel Nano-Crystals by Conjugation of Folic Acid to Pluronic F127: Formulation Optimization, In Vitro and In Vivo Study

**DOI:** 10.3390/molecules27227914

**Published:** 2022-11-16

**Authors:** Nagaraja Sreeharsha, Samathoti Prasanthi, Satyavarapu Veera Venkata Naga Satya Mahalakshmi, Prakash S. Goudanavar, Nimbagal Raghavendra Naveen, Buduru Gowthami, Santosh Fattepur, Girish Meravanige, Syed Mohammed Basheeruddin Asdaq, Md. Khalid Anwer, Bandar Aldhubiab, Mohammed Monirul Islam, Mohammed Habeebuddin, Mallikarjun Telsang, Mazen Al Gharsan, Michelyne Haroun

**Affiliations:** 1 Department of Pharmaceutical Sciences, College of Clinical Pharmacy, King Faisal University, Al-Hofuf 31982, Saudi Arabia; 2 Department of Pharmaceutics, Vidya Siri College of Pharmacy, Off Sarjapura Road, Bangalore 560035, Karnataka, India; 3 Department of Pharmaceutics, Sri Venkateswara College of Pharmacy, RVS Nagar, Tirupati Rd, Chittoor 517127, Andhra Pradesh, India; 4 Department of pharmacognosy, Vishnu Institute of Pharmaceutical Education and Research, Narsapur, Medak 502313, Telangana, India; 5 Department of Pharmaceutics, Sri Adichunchanagiri College of Pharmacy, Adichunchanagiri University, B.G. Nagar 571448, Karnataka, India; 6 Department of Pharmaceutics, Annamacharya College of Pharmacy, New Boyanapalli, Rajampet 516126, Andhra Pradesh, India; 7 School of Pharmacy, Management and Science University, Seksyen 13, Shah Alam 40100, Selangor, Malaysia; 8 Department of Biomedical Sciences, College of Medicine, King Faisal University, Al Hofuf 31982, Saudi Arabia; 9 Department of Pharmacy Practice, College of Pharmacy, AlMaarefa University, Dariyah, Riyadh 13713, Saudi Arabia; 10 Department of Pharmaceutics, College of Pharmacy, Prince Sattam Bin Abdulaziz University, Al-Alkharj 11942, Saudi Arabia; 11 Department of Biomedical Sciences, College of Clinical Pharmacy, King Faisal University, Al Hofuf 31982, Saudi Arabia; 12 Department of Surgery, College of Medicine, King Faisal University, Al Hofuf 31982, Saudi Arabia

**Keywords:** nano-crystals, Paclitaxel, anti-cancer, optimization, central composite design

## Abstract

A brand-new nano-crystal (NC) version of the hydrophobic drug Paclitaxel (PT) were formulated for cancer treatment. A stable NC formulation for the administration of PT was created using the triblock co-polymer Pluronic F127. To achieve maximum entrapment effectiveness and minimal particle size, the formulation was improved using the central composite design by considering agitation speed and vacuum pressure at five levels (coded as +1.414, +1, 0, −1, and −1.414). According to the Design Expert software’s predictions, 13 runs were created and evaluated for the chosen responses. The formulation prepared with an agitation speed of 1260 RPM and a vacuum pressure of 77.53 mbar can meet the requirements of the ideal formulation in order to achieve 142.56 nm of PS and 75.18% EE, according to the level of desirability (D = 0.959). Folic acid was conjugated to Pluronic F127 to create folate receptor-targeted NC. The drug release profile of the nano-crystals in vitro demonstrated sustained release over an extended period. Folate receptor (FR)-targeted NC (O-PT-NC-Folate) has also been prepared by conjugating folic acid to Pluronic F127. MTT test is used to validate the targeting efficacy on the FR-positive human oral cancer cell line (KB). At pharmacologically relevant concentrations, the PT nano-crystal formulation did not cause hemolysis. Compared to non-targeted NC of PT, the O-PT-NC-Folate showed a comparable but more sustained anti-cancer effect, according to an in vivo anti-tumor investigation in NCI/ADR-RES cell lines. The remarkable anti-tumor effectiveness, minimal toxicity, and simplicity of scale-up manufacturing of the NC formulations indicate their potential for clinical development. Other hydrophobic medications that are formulated into nano-systems for improved therapy may benefit from the formulation approach.

## 1. Introduction

One of the fatal diseases in existence today is cancer. In 2020, there will be around 10 million cancer-related deaths worldwide, according to estimates of the disease’s prevalence. By 2030, the WHO predicts a 45 percent rise in cancer-related deaths [1]. Current therapy alternatives, by and large, fall short in the fight against cancer, necessitating the urgent need for new therapeutic tactics, including anti-cancer chemicals and delivery methods. Despite the increased acceptance of oral drugs, intravenous injections remain the most common cancer treatment method [2]. This is mainly because many cancer drugs have poor solubility and/or digestive system toxicity after oral administration, which limits oral absorption below therapeutic levels. More than 40% of newly synthesized therapeutic compounds in research are not water-soluble; hence the problem will not improve [3,4,5]. The proportion of water-insoluble drugs developed in oncology, anti-infective, and anti-viral treatment is even higher [6]. Given the rise in cancer morbidity and the generally poor solubility of many therapeutic substances, approaches for appropriately administering anti-cancer medication are still required, particularly for intravenous administration. Nano-drug delivery methods have received attention due to the improved accumulation of therapeutic drugs based on the enhanced permeability and retention (EPR) effect in malignant tissue [7,8,9,10]. The EPR effect takes advantage of poor tumor vasculature and limited lymphatic drainage, which increases the possibility that nano-particles (NPs) would collect in the tumor [11]. In order to increase the therapeutic efficiency of active pharmaceuticals, numerous nano-carriers, including liposomes, polymer, silica, magnetic, gold, and carbon-based NPs, have been widely used up until now [12,13]. However, due to the small surface area of NPs, these techniques have a relatively low drug-loading capacity [14,15].

The improving drug-loading efficiency, structural stability, steady dissolution, and prolonged circulation duration, the nano-crystallization approach for pharmaceuticals have attracted significant interest in recent years compared to other novel drug delivery systems. Drug nano-crystals (NCs) are sterically stabilized particles having a drug-loading capacity of around 100%. Additionally, they offer extra delivery route options (e.g., oral, parenteral, etc.) [16,17] and have good commercialization potential. Compared to the equivalent amorphous forms, the drug NCs have lesser energy and, as a result, has more structural stability. The crystals’ increased structural stability also causes a slower dissolution. Poorly water-soluble drugs’ solubility and dissolution rate increase as drug NCs’ size reduces [18,19].

The US Food and Drug Administration has approved the powerful anti-neoplastic drug paclitaxel (PT), which is effective against a variety of cancers, including non-small cell lung cancer, breast cancer, and ovarian cancer [20]. Abraxane ^TM^, which comprises 10% (w/w) PT and 90% (w/w) albumin, was recently given FDA approval for cancer treatment. This formulation is the first commercially available Cremophor-EL-free PT formulation, and it was produced by high-pressure homogenization of a colloidal nano-particle PT solution in presence of serum albumin. Abraxane ^TM^ offers several benefits over the common PT/Cremophor-EL combination, many of which reduce the toxicities often connected with PT-based therapy [21,22].

Abraxane’s cost, several thousand dollars per dose, keeps it from frequently taking the place of Taxol in oncological treatment. Pharmaceutical companies are under more pressure than ever to lower healthcare costs while still adding value for patients and healthcare providers. When creating novel nano-particle compositions, it is essential to keep production costs as low as possible. Nano-drug carriers typically have a low drug-to-carrier ratio, making it challenging to improve drug-loading efficiency. Numerous efforts have been made to improve the amount of medicine the body can absorb.

An ideal nano-particle should possess a high ratio of the carried drug to excipients, particularly for nano-particles designed to target cancer cells. It would be advantageous if a large amount of drug is delivered for each targeting event. This study addresses the challenges of improving the drug-loading efficiency and decreasing the high production costs of nano-particles. Additionally, the current research work focused on synthesis of folate-conjugated F127 and its application in production of the folate receptor-targeted NC in enhancing anti-tumoral properties. 

## 2. Materials and Methods

Pluronic F127 was purchased from Sigma Aldrich (St. Louis, MO, USA), while PT and Taxol were purchased from Yarrow Chem, Mumbai, India and. All further compounds are of the analytical variety. NCI/ADR-RES, a resistant human ovarian cancer cell line, was obtained from National Centre for Cell Science (NCCS) in Pune, Maharashtra, India. Five-week-old female BALB/c nude mice were bought from National Institute of Nutrition, India. The institutional animal ethics committee’s guidelines were followed for all in vivo experiments on animals (Approval Number-SACCP-IAEC/2022-01/54).

### 2.1. Compatibility Studies [23,24]

#### 2.1.1. Fourier Transform Infrared Spectroscopy (FTIR)

Using KBr-pressed discs in the 400–4000 cm^−1^ range at room temperature, FTIR spectra of drugs, excipients, and physical combinations were captured.

#### 2.1.2. Differential Scanning Calorimetry (DSC)

The DSC curves were obtained using a DSC Q20 (TA instrument) and a dynamic nitrogen atmosphere with a flow rate of 50 mL min^−1^. We placed 2 mg of samples in a sealed aluminum pan [25,26]. The analysis was conducted between 30 and 450 °C at a heating rate of 10 °C min^−1^. The DSC cell was calibrated using indium (156.6 °C). DSC curves were analyzed using TA Instrument Universal Analysis program.

### 2.2. Preparation of PT-NC Formulation 

The PT-NC was produced via anti-solvent precipitation, and the process parameters were enhanced using the response surface methodology combined with Central Composite Design (CCD). In a glass vial, 2.5 mg of PT were dissolved in 1.5 mL of 100% methanol, then 10 mL of water was slowly added while stirring (900–1500 rpm). The solubilized drug was precipitated by rota vaporizing the entire solution to a pressure of 40–80 mbar, reaching that pressure for 30 min, and then reducing it to 10 mbar. To eliminate any remaining solvent, the resultant powder was held under a vacuum [27]. 

The dry powder was hydrated with an F127 aqueous solution after a 10 min sonication to form the NC suspension [PT-NC-S]. We ensured 2.5 mg PT and 5 mg F127 were always included in the final NC formulation. Thus, NC suspensions prepared with this API/stabilizer ratio were used in the in vitro and in vivo investigations. However, varied water volumes of redispersion were used depending on the application to yield 0.08 percent weight volume percentages of F127. Significant PT concentrations were correctly adjusted for the associated control trials.

#### Experimental Design

The development of PT-NC is standardized using the statistical model RSM. The decoded values for the independent parameters agitation speed (X1) and vacuum pressure (X2) are −1.414 (low), −1, 0 (medium), +1, and +1. (high). Using Design Expert Version 12 (Stat Ease Inc., Minneapolis, MN, USA), 13 experiment runs were created to examine these factors’ effects on entrapment efficacy [EE] and nano-crystal size [PS]. The whole experiment plan is shown in Table 1, along with examples of coded and actual values for the selected parameters and restraints for the selected answers. To validate the created polynomial equations, analysis of variance (ANOVA) was utilized [28,29]. All test runs also used a variety of statistical approaches to choose the model that suited the data the best. Each test run used a quadratic design to quantify the response and regression analysis of the outcome.

(1) $$Y_{i\left(\mathrm{Quadratic}\right)}=b_{0}+b_{1}X_{1}+b_{2}X_{2}+b_{3}X_{3}+b_{4}X_{1}X_{2}+b_{5}X_{1}X_{3}+b_{6}X_{2}X_{3}+b_{7}X_{1}^{2}+b_{8}X_{2}^{2}+b_{9}X_{3}^{2}$$

where

Y_i_—chosen response or dependent variable;

b_o_—computed response;

b_i_—the estimated coefficient for main effects (X_1_, X_2_, X_3_); interaction terms of main effects (X_1_X_2_, X_2_X_3_, X_1_X_3_), and polynomial terms of independent variables (X_1_^2^, X_2_^2^, X_3_^2^).

- PS

Using the dynamic light scattering method, Malvern Zetasizer investigated the PT-NC average PS, poly-dispersity index (PDI), and electrokinetic potential (2000, UK) [30]. To prevent the blockage of particles, the prescribed amount of VS-NC was re-dispersed into a generous amount of milli-Q water and vortexed for 5 min. At 25 °C, the final sample was evaluated in triplicate for 1 min.

- EE

To ascertain the success of PT’s encapsulation, the NC was maintained at room temperature for 1 h, fed into 0.45 and 0.22 mm centrifugal filter devices and a 3000 MWCO micron, respectively, and centrifuged at 16,000 g for 20 min. According to Feng Liu et al., HPLC measured PT in NC and free PT in the filtrate [20].

### 2.3. Preparation of Optimized Formulation 

An optimized formulation of PT-NC was prepared with the conditions as calculated using the design expert program and characterized.

### 2.4. PT-NC Morphology

NCs’ morphological evaluation was looked at using a scanning electron microscope (SEM) (Philips XL 30 microscope, Hillsboro, OR, USA). Processed PT-NC powder was applied to a double-sided tape, coated with a 30 nm coating of gold, and then subjected to a 2 min period of vacuum (10-6 Pa), and SEM observations at a 15 kV accelerating voltage [29] were observed at working distance of 15.2 mm using T detector.

### 2.5. Dissolution Study

To ascertain the release kinetics in dialysis cassettes, the PT-NC-S formulation was diluted in 400 mL of dialysis buffer (Phosphate buffer solution-(pH 7.4) with 0.05 percent Tween 80) (molecular weight threshold 10,000 Da). For control, Taxol, the pharmaceutical form of PT in Cremophor EL, was mixed with dehydrated USP-grade ethanol (1:1). Samples of dissolution media were taken every two minutes and replaced with the same volume of fresh dialysis buffer at predetermined intervals. Changing the entire dialysis buffer at pre-determined intervals was necessary to keep the sink condition. One micro-gram of n-butyl p-hydroxy benzoate (10 µL) was added to the aliquots as an internal standard liquid (STD). A liquid extraction extracted PT and STD with methyl tert-butyl ether. For HPLC analysis, the residue was dispersed in an 80/20 mixture of water and methanol, and the nitrogen stream was utilized to evaporate it. The HPLC system employed a 25 mm, 4.6 mm reverse phase C18 column, a UV detector, and a flow rate of 1 mL/min at 228 nm [31,32].

### 2.6. Short-Term Stability Studies

A short-duration stability test was used to investigate the stability of PT-NC-S. A rubber cork stopper was used to close the amber glass bottles containing NC [33]. As soon as this was completed, the glass bottles were placed into stability chambers that were kept at 40 °C, and a 60 °C and a 75 % relative humidity were maintained for around six months. Test samples’ physical properties (PS and PDI) were analyzed at different time intervals [34,35].

### 2.7. Production of the FR-Targeted NC and Conjugation of Folic Acid to Pluronic F127

**Figure 1:**
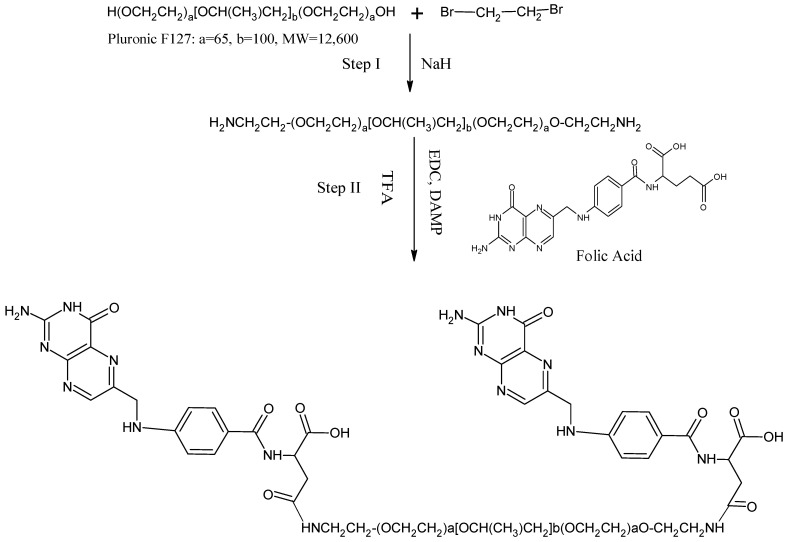
Schematic representation of conjugation of folic acid to Pluronic F127 nitrogen as the reagent.

Folate-conjugated F127 was synthesized in two steps (see Figure 1). Step 1 was the addition of an amino function to the ends of the Pluronic: a 100 mL three neck round bottom flask was fitted with a nitrogen balloon, and a mixture of anhydrous chloroform (50 mL), Pluronic (1.26 g), sodium hydride (NaH) (0.24 g), and 2-bromoethylamine hydrobromide (0.20 g) were added and allowed to react for 48 h at room temperature. After completion of the reaction, excess NaH in the reaction mixture was quenched by methanol (2.03 mL). The modified Pluronic was purified by dialysis against de-ionized water, followed by lyophilization. In the second stage of the process, folic acid was mixed with modified Pluronic (0.28 g), amino Pluronic (0.19 g), 1-ethyl-3-(3-dimethylaminopropyl) carbodiimide hydrochloride (EDC, 0.42 g), 4-dimethyl aminopyridine (DMAP, 0.03 g), triethylamine (0.06 mL), and N-nitroso folate A size exclusion column was utilized for the chromatographic analysis of the reaction mixture [31]. The recovered F127-folate-containing empty volume eluent was lyophilized after being dialyzed (with a membrane more significant than 10,000 Da) against de-ionized water. In order to produce the FR-targeted NCs, PT was first dissolved in chloroform, followed by the addition of different quantities of F127 and F127-folate. The selected NCs were subjected to the same process as before [31]. 

### 2.8. Evaluation of the Targeted NC in an FR-Positive Tumor Cell Line

The MTT assay was carried out to determine the level of cytotoxicity exhibited by the targeted NCs in a FR-positive human oral cancer cell line (KB), in the same manner as was previously described, with only a few minor adjustments [36]. A folate-free DMEM medium containing 5% fetal bovine serum was used to cultivate the KB cell line as a mono-layer for five weeks. After being briefly suspended with 10 mM EDTA, the cells were transplanted onto 96-well tissue culture plates with 1 × 10^4^ cells per well 24 h before the administration of the drug addiction [37]. The culture media were then swapped out with 100 mL of the standard medium that included successive dilutions of test samples. The cells were cultivated in 200 mL of fresh media after being incubated for the first two hours at 37 °C. After 48 h of incubation, the MTT assay was carried out.

### 2.9. Evaluation of Hemolytic Activity

The cytotoxicity of the targeted NCs was tested in an FR-positive human oral cancer cell line using the MTT test, as described [38]. KB cell line was cultured in DMEM with 5% FBS and no folate for five weeks. Twenty-four hours before drug addiction, 1 × 10^4^ cells were transplanted onto 96-well tissue culture plates with 10 mM EDTA. Then, 100 mL of standard medium with O-PT-NC and O-PT-NC-CEL was added. After two hours at 37 °C, cells were cultured in 200 mL of the new medium. After 48 h, MTT was performed.

### 2.10. Animals and Materials

Five-week-old female BALB/c nude mice were bought from the National Cancer Institute (NCI).

#### 2.10.1. In Vivo Anti-Cancer Activity

A total of 5 × 10^6^ NCI/ADR-RES cells were injected subcutaneously into the right flanks of nude mice to perform the inoculation. After the tumor mass in the xenograft had formed, random assignments of mice to treatment groups with a total of five mice in each group were carried out. Mice were then intravenously injected with various PT formulations (O-PT-NC and O-PT-NC-Folate) at 10 mg/kg for every single dose [20]. Treatments were administered on days 0, 3, 6, 9, and 12. The tumor volume was measured and estimated every other day as (length × width^2^)/2. When the tumor’s long dimension measured 2 cm, mice were slaughtered. 

#### 2.10.2. Statistics

The mean and standard deviation of the data are displayed. In animal research, significance among groups was determined using a one-way analysis of variance (ANOVA). In both the stability and the cytotoxicity investigations, a two-tailed Student t-test was utilized for the purpose of statistical analysis. A value of *p* less than 0.05 was considered significant for the data.

## 3. Results and Discussion

### 3.1. Compatibility Studies

**Figure 2:**
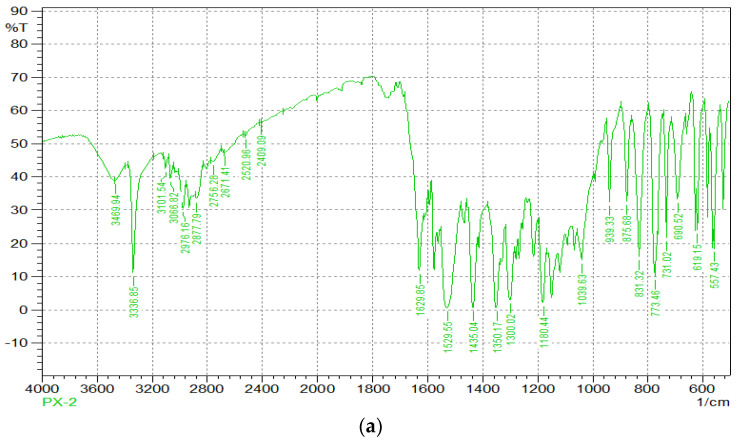

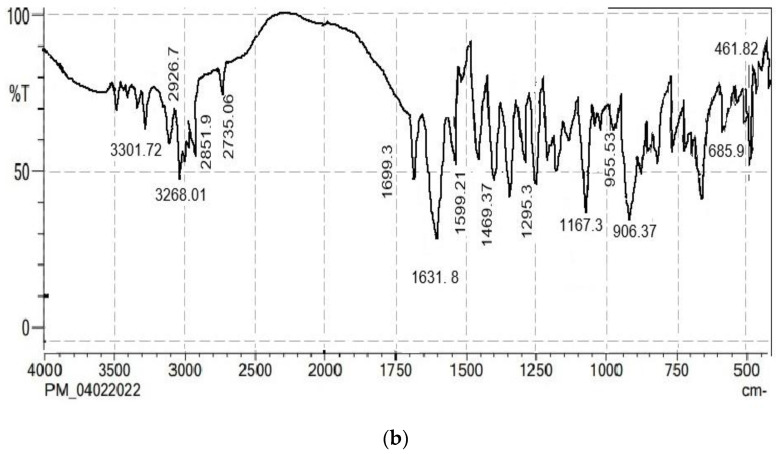
FTIR Spectra of (**a**) PT and (**b**) physical mixture of PT and polymers.

Figure 2 depicts the FTIR spectra of PT and a physical mixture of PT and polymers. Characteristic peaks for -NH stretching, C-N stretching, and C=O stretching, respectively, are located at 3336.85, 1180, and 1629.85 cm^−1^. The range 941–831 cm^−1^ further confirms the C-H in-plane distortion. O-H stretching is represented by 3336–2500 cm^−1^. The distinctive peaks at 2973–2541 cm^−1^ and 1652-1579 demonstrate CH_3_/C-H asymmetric stretching and C-C stretching. The FTIR spectra of PT with polymers were used to identify the aforementioned distinctive groups. This appears to indicate no chemical interaction between the polymer and the pharmaceuticals in the micro-spheres and that the drugs are present in the polymer matrix as a molecular dispersion.

The DSC thermogram of PT and a physical mixture of PT and polymers were obtained to assess the thermal behavior (Figure S2). The endothermic peak at 216 °C, which corresponds to the melting point of 216–217 °C, defines PT. Physical mixtures have also shown a similar endothermic peak with a slight variation (at 214 °C), supporting the unchanged thermal behavior of PT with particular polymers.

### 3.2. Optimization 

The effect of particular variables and how they interacted to produce the minimal PS and greatest EE were studied using CCD. A total of 13 experimental trials in total were anticipated, and Table 2 lists the observed results. Experimental formulations’ PS was located in the nm range between 98 and 315. EE varied from 58 to 96 percent, which was be used to calculate how much drug had been entrapped. All experimental results were analyzed for specific reactions using the fx model and the ANOVA.

The quadratic model was selected for all responses after performing a sequential sum of squares (Type-I) and a fit summary. The model was selected after considering the F-value, *p*-value, and R^2^ values of the model. Table 3 shows that the quadratic model has the largest polynomial order, and its *p*-value, which measures the degree of significance, is 0.0001.

The deviation of less than 0.2 between the predicted R^2^ value of 0.8587 and the adjusted R^2^ value of 0.9632 can be seen for EE. The signal-to-noise ratio is something that Adeq Precision measures. A ratio of at least four is preferred. A strong signal is indicated by the ratio of 27.8130. To move around the design space, utilize this model. For PS [0.8435, 0.9582, and 19.8857] [39], similar outcomes were seen. The normal plot of residuals [40] provided additional evidence of all these chosen models’ correctness. The visual inspection graph is satisfactory; thus, the prescribed statistical program will not be used for this. The proposed model can be accepted statistically because all studentized residuals for the selected responses were distributed closer to the straight line [25,26,41]. Figure S1, in Supplementary Materials, denotes the experimental test against the residuals to identify the hidden factors influencing the answers. A time-coupled variable in the background was indicated by observing a scattered trend within the acceptable range. The value of the coefficient of variation, often known as CV, is one method that may be utilized to demonstrate that the repeatability of an experiment guarantees reliable results and enables transparent comprehension of the process. The consistency and accuracy of the design were ensured because the required CV value was lower than the prescribed (CV-10%) value (2.49 % for EE and 8.48 % for PS). Lack of fit is an additional parameter that assesses how well the model captures all data [33]. The ANOVA findings clearly show that the lack of fit is non-significant (*p* > 0.05), which supports the fitness of the chosen design. An ANOVA was used to investigate the quantitative impacts of particular factors on replies. Multiple regression was applied to the collected data to produce polynomial equations. All selected models are likely to be significant based on the model F-values of 63.84 and 56.05.

For EE, A, B, AB, A2, and B2 are basic significant model terms. The experimental layout suggested that the following factors could have an impact on EE: (i) antagonistic effects of factor A and polynomial terms of A and B, with *p*-values of 0.0041, 0.0001, and 0.0032, respectively; and (ii) synergistic effects of B, with AB effects being the highest of all the terms. According to the experimental plan, PS might be impacted by two factors: (i) the antagonist action of factor B; and (ii) the synergistic effect of polynomial terms of A and B, with B impacts being the more significant (see Table 4).

Equations generated for coded factors:

EE = +88.20 − 1.41 A + 8.15 B + 11.25 AB − 5.91A^2^ − 3.41 B^2^ (2)

PS = +118.00 + 9.42 A − 65.05 B + 13.50 AB + 34.94 A^2^ + 50.19 B^2^ (3)

**Figure 3:**
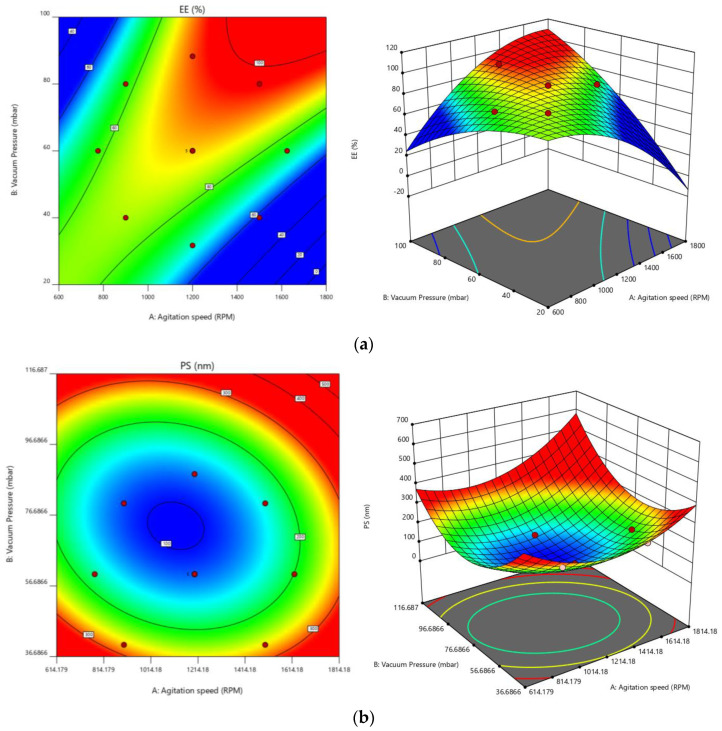
Contour plots and three-dimensional response surface graphs for (**a**) EE and (**b**) PS.

Any given concentration of the chosen elements can be predicted using any of the abovementioned equations. In addition, factor coefficients help determine each component’s relative influence on the replies. These graphs are used to represent the measured responses, and contour plots and three-dimensional response surface graphs (also known as RSG) are crucial for explaining the interaction and the main effect (see Figure 3).

**Figure 4:**
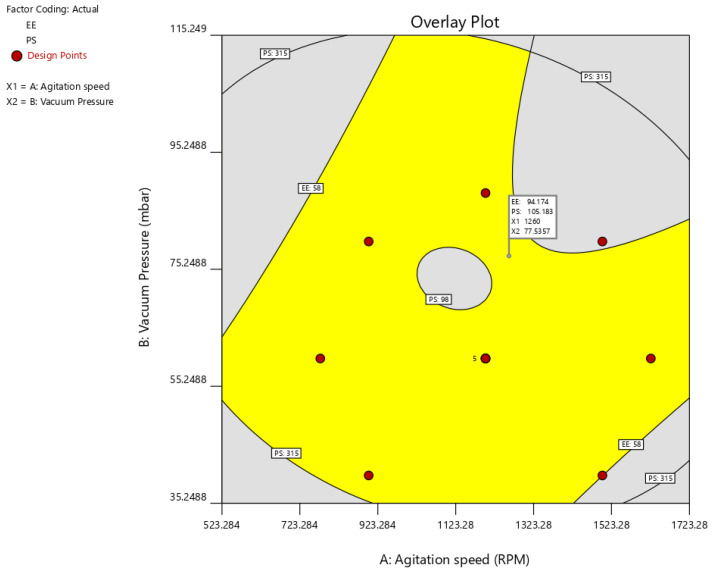
Overlay plot of optimization result.

Using the desirability function (D), it is possible to optimize various models acquired through the experimental study. To produce the overlay graph, several restrictions, including particle size, zeta potential maximum, and PDI minimum, were specified for each response [42,43]. The design space included every one of the chosen variables. The highest D value of 0.959 for all the responses’ combined desirability plots was reached at the best independent variable concentrations. The critical responses were overlaid in the contour plot (see Figure 4). A formulation created utilizing an agitation speed of 1260 RPM, and a vacuum pressure of 77.53 mbar, can achieve the requirements of the optimum formulation based on this desirable technique. Therefore, these optimized concentrations can obtain an EE of 94.17 with 105.19 nm PS. An optimized formulation of O-PT-NC was created and assessed using these projected optimal concentrations. The experimental results were compared with theoretical values so that the findings could support the experience design. A relative error of less than 2 percent was found, providing conclusive evidence that the design was accurate.

### 3.3. Surface Morphology

**Figure 5:**
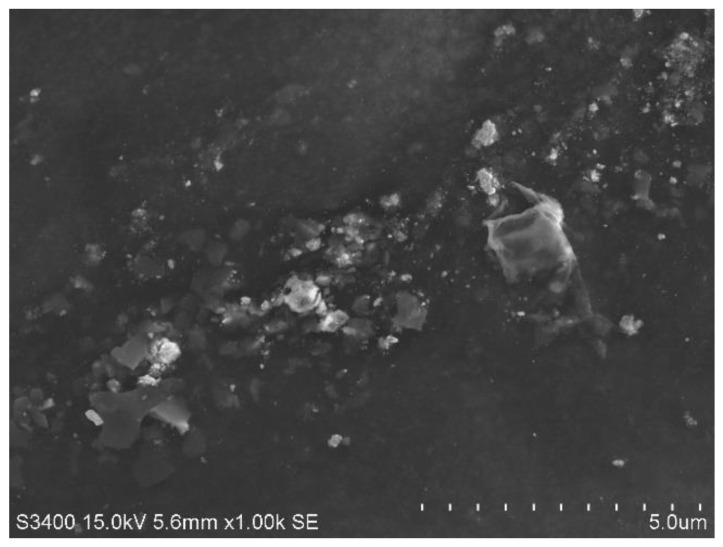
SEM image of O-PT-NC.

SEM characterized the surface morphology to establish the size and morphology of O-PT-NC. With sizes ranging from 80 to 120 nm, naked PT nano-crystals displayed a rod-like structure (see Figure 5) and retained their rod-like form despite being longer. The relative length of nano-crystals is expected to decrease renal clearance and increase plasma residence time. Both O-PT-NC and O-PT-NC-Foliate show similar particle size distribution (Supplementary Materials File S1). 

### 3.4. Drug Release Study

**Figure 6:**
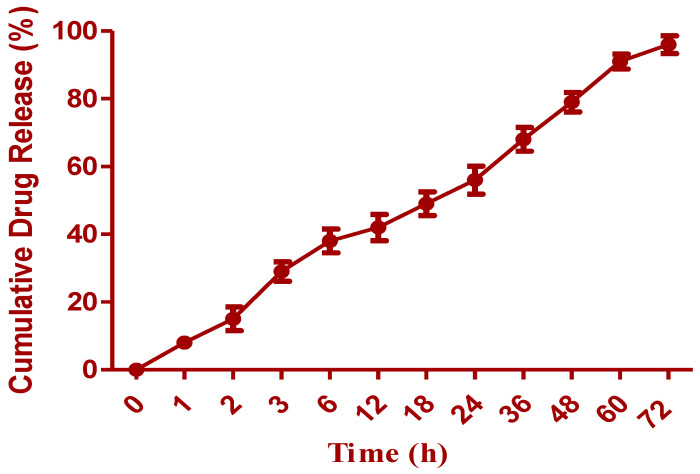
In vitro dissolution profile of optimized formulation of PT.

To ascertain whether PT would be released from nano-crystals prior to cellular absorption, the in vitro drug release profile of O-PT-NC was examined. Figure 6 shows that nano-crystals exhibit a significantly controlled release profile. Optimized nano-crystal formulation resulted in a cumulative release of PT of roughly 96.45 percent after 72 h. PT was trapped in Cremophor micelles while under Taxol, resulting in a quick release (100 percent within 22 h). Since nano-crystals did not undergo an initial burst release of PT, it was not anticipated that PT would be located on or close to the surface of the nano-crystals. Instead, the nano-crystals encased PT in a shell. This notion was supported by evidence that was provided by an X-ray photo spectrometer (XPS). In order to prove that F127 was successfully applied to the surface of PT, a specific scan was performed on the nitrogen, which can only be found in PT molecules. The absence of a nitrogen signal proved the coating. Because there is no surfactant coating on the surface, it makes sense that pure PT crystals showed a relatively quick release.

### 3.5. Stability Studies

Throughout the stability testing period, there was no change in the NC’s appearance in either of the storage settings. No significant changes were identified in PS and PDI under all the storage conditions, confirming the formulation’s stability (see Table 5).

### 3.6. Folate Receptor-Targeted Nano-Crystals (O-PT-NC-Folate)

**Figure 7:**
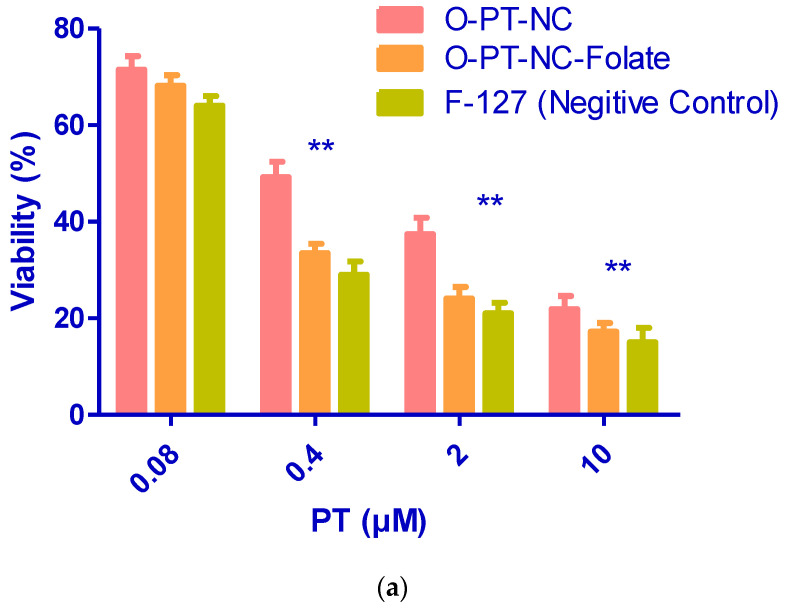

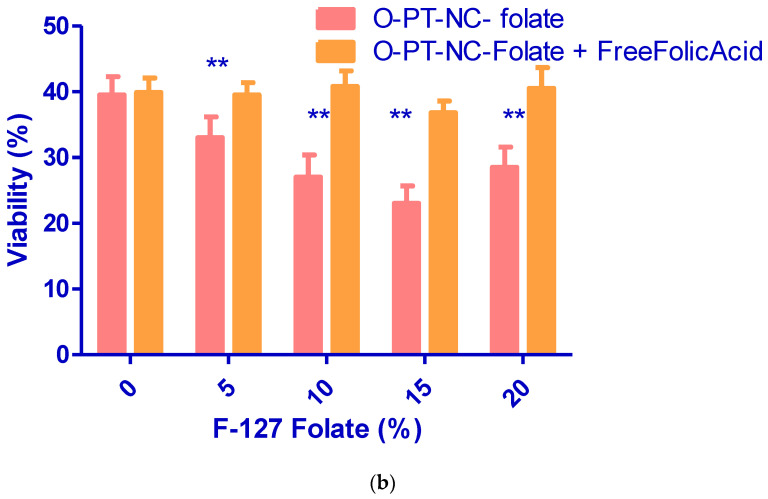
Viability of the human FR-positive oral cancer cell line in the presence of the targeted (O-PT-NC-Folate) and non-targeted (O-PT-NC) nano-crystals following a 48 h incubation period. The targeted nano-crystals comprised (**a**) varying levels of F127-folate with different PT concentrations and (**b**) diverse concentrations of F127-folate with 2 mM PT. Untreated cells were used as a control group, and their vitality was measured at 100%. The data were provided as the mean and standard deviation (n = 8). ** *p* < 0.01.

The efficacy and toxicity of anti-cancer medications can both be improved by selectively targeting the tumor. Therefore, much work has gone into creating targeted nano-particles, which are created by adding targeting molecules to the surface of the nano-particles, such as oligonucleotides, peptides, and antibodies. The folate receptor is an attractive target for the delivery of anti-cancer drugs since it is overexpressed in many cancerous tumors. Folic acid was coupled to Pluronic F127, and the same procedure was used to synthesis folate receptor-targeted NCs. The NCs targeted folate receptors. MTT assay was used to compare FR-targeted and non-targeted NCs in FR-positive KB cells. When the concentration of PT was increased from 0.08 to 10 mM, as shown in Figure 7a, the viability of targeted NCs that contained 10% F127-folate was considerably lower (*p* 0.001) than the vitality of non-targeted NCs (O-PT-NC). Figure 7b demonstrates that cytotoxicity decreased from 10% to 5% F127-folate. F127-folate concentrations of greater than 10% did not increase cytotoxicity. FR-specific cytotoxicity of FR-targeted NCs was revealed by removing differential cytotoxicity in the presence of 1 mM free folate.

### 3.7. Hemolytic Activity

Pluronic F127 is a desirable choice for building a pharmaceutical vehicle to deliver due to its low toxicity and bio-compatibility of medications through various delivery methods. It is believed that PT/Cremophor-EL will be far more dangerous than the PT NCs made here because there was very little F127. The toxicity of the NCs was first determined by observing their effects on mice to establish the highest safe dose. When the dose of PT in Cremophor-EL was increased to 30 mg/kg, six out of the six injected animals died. When the dose of PT NCs was increased, however, none of the six mice that were injected with PT NCs died, even when the dose was increased to as high as 60 mg/kg. Female nude mice weighing 18–20 g were injected through the IV route with PT in Cremophor-EL.

**Figure 8:**
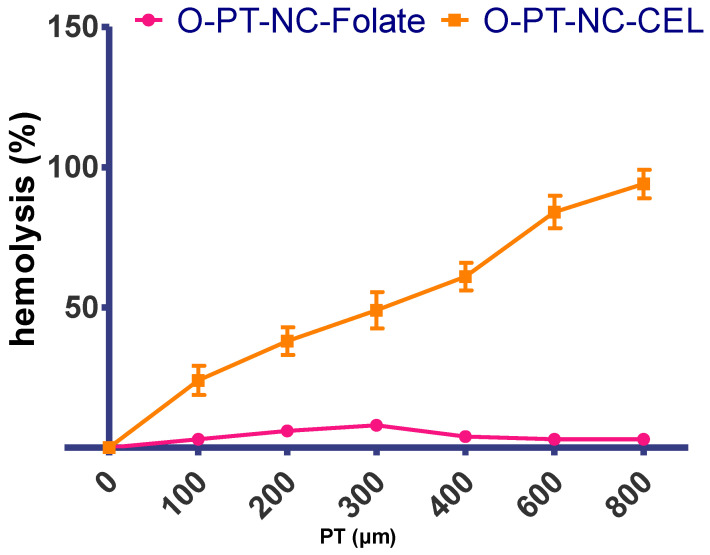
Hemolysis activity of the O-PT-NC-Folate and O-PT-NC-CEL. There was evidence of hemolytic activity in the erythrocyte suspensions collected from mouse blood (n = 3, mean SD).

It is well-known that surfactants trigger hemolysis in red blood cells. Due to the significant amount of surfactant used in the formulation of commercial Cremophor-EL, PTs hemolytic properties have already been described (polyoxyethylated castor oil) [44]. The formulation’s hemolytic potential was assessed to determine the safety of the NCs for intravenous (IV) administration. The quantity of membrane damage a drug or its formulation will cause when people consume it can be quickly and accurately predicted using drug-induced hemolysis in vitro [45]. The range of concentrations that were looked at was 0–0.8 mM. Figure 8 shows the activity of the O-PT-NC-Folate and O-PT-NC-CEL (O-PT-NC-Cremophor-hemolytic EL) in a suspension of mouse erythrocytes. At a PT dose of 0.2 mM, the NCs were much less harmful to the blood than the PT/Cremophor-EL. When 20 mg/kg of PT was given intravenously to a 20 g mouse, the amount of the drug in its blood was about 0.30 mM. (the blood volume is estimated to be 7.3 percent of the body weight for mice) [46,47].

Figure 8 shows that Cremophor-EL did not affect NCs, but a PT dose of about 300 mM caused about 50% of the blood cells to break down. When the amount of F127 went up to 0.8 mM, there was no hemolysis in the O-NT-NC, but there was full hemolysis in the O-PT-NC-CEL. When all the results were looked at together, they showed that formulated PT-NC-Folate formulation was much safer than Cremophor-EL-loaded PT formulation [48].

### 3.8. In Vivo Anti-Cancer Effects

**Figure 9:**
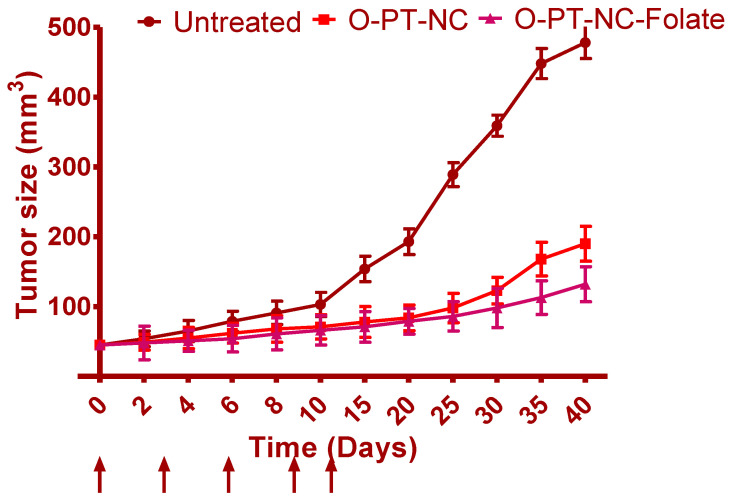
Tumor growth inhibition effect of O-PT-NC and O-PT-NC-Folate in the NCI/ADR-RES xenograft model. (Solid arrows indicate the days of intravenous administration.)

Figure 9 shows the treatment effectiveness after using different formulations on NCI/ADR-RES xenografts in naked mice. After the research, the tumor size in untreated mice was 478.54 mm^3^ and continued to grow significantly for another 40 days. However, rats given 10 mg/kg of O-PT-NC showed apparent tumor regression. A greatly strengthened tumor inhibition effect can be seen by the highly slight change in tumor size, which only increased to 190.54 mm^3^. Additionally, O-PT-NC-Folate had a comparable but more potent anti-cancer impact than O-PT-NC. Throughout the investigation, the mice’s body weight was also recorded. The mice treated with either formulation did not lose weight indicating that the PT formulations were well tolerated.

## 4. Conclusions

We investigated the NCs of PT can be successfully created using a simple process utilizing F127 as the only excipient. The response surface methodology, in conjunction with several other statistical estimations, was utilized throughout the development of the PT-NC to maximize the numerous process factors contained within the formulation. In a nutshell, the findings of this study lend credence to the hypothesis that new NCs can function effectively as a drug delivery vehicle for cancer treatment. Because of the benefits mentioned above, this formulation has a commercialization potential that could be realized. To begin, the NCs were successful in achieving maximal drug-loading efficiency. Second, the procedure for manufacturing the NCs is straightforward and does not include any alterations to the drug’s chemical composition or the excipient. The procedure is quite amenable to being scaled up for industrial use. In addition, the optimized approach is thought to be appropriate for formulating additional hydrophobic medications, such as NCs, for treating various disorders. Additionally, the NCs can be further modified by conjugating folic acid for FR targeting to boost the therapeutic efficacy of anti-cancer medications and facilitate targeted delivery of these drugs. Further hemolytic and in vivo anti-cancer studies demonstrate that formulated and targeted NC is beneficial in treating malignancies. This nano-scaled formulation might offer a fresh approach to the age-old problem of how to treat cancer patients who have become resistant to multiple drugs.

## Figures and Tables

**Table 1:** Experimental plan for CCD in terms of actual and coded values.

Factors/Independent Variables	Levels					Responses/Dependent Variables	Constraints
	−1.414	−1	0	+1	+1.414		
The agitation speed—X_1_	775.736	900	1200	1500	1624.26	EE	Maximum
Vacuum Pressure—X_2_	31.7157	40	60	80	88.284	PS	Minimum

**Table 2:** Experimental runs projected and their responses observed.

		Factor 1	Factor 2	Response 1	Response 2
Std	Run	A:Agitation speed	B:Vacuum Pressure	EE	PS
		RPM	mbar	%	nm
7	4	1200	31.72	69	315
1	8	900	40	84	265
2	12	1500	40	58	258
5	1	775.736	60	79	187
11	2	1200	60	88	110
12	7	1200	60	87	114
9	9	1200	60	89	116
10	10	1200	60	88	125
13	11	1200	60	89	125
6	13	1624.26	60	76	212
3	5	900	80	75	98
4	6	1500	80	94	145
8	3	1200	88.28	96	145

**Table 3:** Model statistical summary.

Response	Models	R^2^	Adju.R^2^	Pred.R^2^	Adequate Precision	Sequential *p*-Value	Remarks
EE	Linear	0.3977	0.2772	−0.2036	----	0.0793	
	2 FI	0.7658	0.6877	0.3704	27.8130	0.0045	
	Quadratic	0.9785	0.9632	0.8587	---	0.0002	Suggested
	Cubic	0.9906	0.9774	0.5256	---	0.1270	Aliased
PS	Linear	0.5762	0.4915	0.3042	---	0.0022	
	2 FI	0.5884	0.4512	0.2250	---	0.0137	
	Quadratic	0.9756	0.9582	0.8435	19.8857	<0.0001	Suggested
	Cubic	0.9789	0.9495	−0.1583	---	0.6942	

**Table 4:** Analysis of variance (ANOVA) results.

	Intercept	A	B	AB	A²	B²
**EE**	88.2	−1.40533	8.14797	11.25	−5.9125	−3.4125
***p*** **-values**		0.0041	<0.0001	<0.0001	0.0001	0.0032
**PS**	118	9.41942	−65.052	13.5	34.9375	50.1875
***p*** **-values**		0.1078	<0.0001	0.1039	0.0004	<0.0001

**Table 5:** Stability studies for O-PT-NC.

TEST	INITIAL	25 °C ± 2 °C + 60% ± 5% RH		40 °C ±2 °C + 75% ±5% RH	
		3 M	6 M	3 M	6 M
Physical characteristics	Complies	Complies	Complies	Complies	Complies
PS	106.21	105.78	104.24	104.34	103.52
PDI	0.18	0.19	0.20	0.20	0.21

## Data Availability

The data presented in this study are available in supplementary material.

## Supplementary Materials

The following supporting information can be downloaded at: https://www.mdpi.com/article/10.3390/molecules27227914/s1, Figure S1: Particle size distribution of (a) O-PT-NC and (b) O-PT-NC-Foliate; Figure S2: DSC thermogram of (a) Pure PT and (b) O-PT-NC.
