# Supplementary material for: Enhancement of Anti-Tumoral Properties of Paclitaxel Nano-Crystals by Conjugation of Folic Acid to Pluronic F127: Formulation Optimization, In Vitro and In Vivo Study
Source: Molecules. 2022 Nov 16;27(22):7914. doi: 10.3390/molecules27227914 (PMC9696646; doi:10.3390/molecules27227914)
Supplement: Supplementary file 1 [file molecules-27-07914-s001.zip › molecules-1975912-supplementary.pdf]

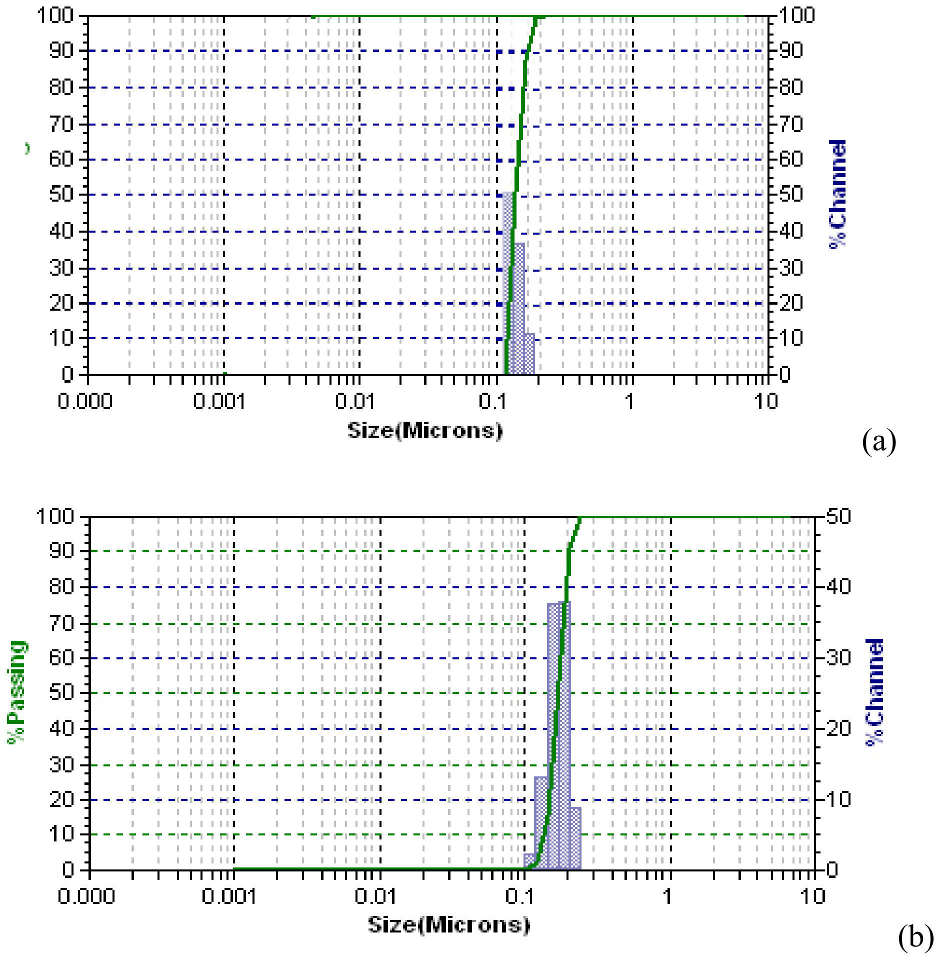

Figure S1. Particle size distribution of (a) O-PT-NC and (b) O-PT-NC-Foliate.

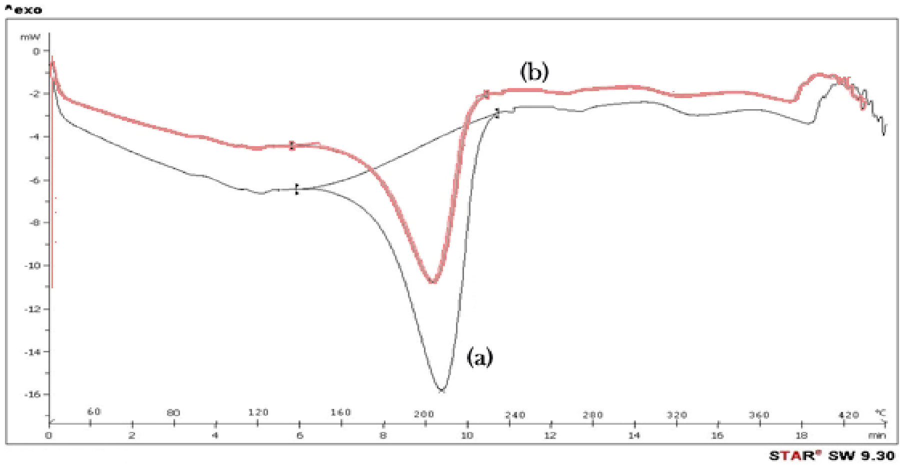

Figure S2. DSC thermogram of (a) Pure PT and (b) O-PT-NC.
